# High-Dose Vitamin C for Cancer Therapy

**DOI:** 10.3390/ph15060711

**Published:** 2022-06-03

**Authors:** Ali Mussa, Ros Akmal Mohd Idris, Naveed Ahmed, Suhana Ahmad, Ahmad Hafiz Murtadha, Tengku Ahmad Damitri Al Astani Tengku Din, Chan Yean Yean, Wan Faiziah Wan Abdul Rahman, Norhafiza Mat Lazim, Vuk Uskoković, Khalid Hajissa, Noor Fatmawati Mokhtar, Rohimah Mohamud, Rosline Hassan

**Affiliations:** 1 Department of Haematology, School of Medical Sciences, Universiti Sains Malaysia, Kubang Kerian 16150, Kelantan, Malaysia; alimaha1341989@gmail.com; 2 Department of Biology, Faculty of Education, Omdurman Islamic University, Omdurman 14415, Sudan; 3 Department of Immunology, School of Medical Sciences, Universiti Sains Malaysia, Kubang Kerian 16150, Kelantan, Malaysia; rosakmalmohdidris@gmail.com (R.A.M.I.); suhanaahmad1207@gmail.com (S.A.); 4 Department of Medical Microbiology and Parasitology, School of Medical Sciences, Universiti Sains Malaysia, Kubang Kerian 16150, Kelantan, Malaysia; namalik288@gmail.com (N.A.); yychan@usm.my (C.Y.Y.); khalid541983@yahoo.com (K.H.); 5 Institute for Research in Molecular Medicine (INFORMM), Universiti Sains Malaysia, Health Campus, Kubang Kerian 16150, Kelantan, Malaysia; hafizmurtadha95@gmail.com (A.H.M.); fatmawati@usm.my (N.F.M.); 6 Department of Chemical Pathology, School of Medical Sciences, Universiti Sains Malaysia, Kubang Kerian 16150, Kelantan, Malaysia; damitri@usm.my; 7 Department of Pathology, School of Medical Sciences, Universiti Sains Malaysia, Kubang Kerian 16150, Kelantan, Malaysia; wfaiziah@usm.my; 8 Department of Otorhinolaryngology, School of Medical Sciences, Universiti Sains Malaysia, Kubang Kerian 16150, Kelantan, Malaysia; norhafiza@usm.my; 9 TardigradeNano LLC., Irvine, CA 92604, USA; vuk.uskokovic@tardigradenano.com; 10 Department of Mechanical Engineering, San Diego State University, San Diego, CA 92182, USA

**Keywords:** high-dose, anti-cancer, vitamin-C, pharmacological, pharmacokinetics, cancer, immunotherapy, ROS-scavenging systems

## Abstract

In recent years, the idea that Vitamin C (Vit-C) could be utilized as a form of anti-cancer therapy has generated many contradictory arguments. Recent insights into the physiological characteristics of Vit-C, its pharmacokinetics, and results from preclinical reports, however, suggest that high-dose Vit-C could be effectively utilized in the management of various tumor types. Studies have shown that the pharmacological action of Vit-C can attack various processes that cancerous cells use for their growth and development. Here, we discuss the anti-cancer functions of Vit-C, but also the potential for the use of Vit-C as an epigenetic regulator and immunotherapy enhancer. We also provide a short overview of the current state of systems for scavenging reactive oxygen species (ROS), especially in the context of their influencing high-dose Vit-C toxicity for the inhibition of cancer growth. Even though the mechanisms of Vit-C action are promising, they need to be supported with robust randomized and controlled clinical trials. Moreover, upcoming studies should focus on how to define the most suitable cancer patient populations for high-dose Vit-C treatments and develop effective strategies that combine Vit-C with various concurrent cancer treatment regimens.

## 1. Introduction

Vit-C (C_6_H_8_O_6_)-ascorbate or/and ascorbic acid (Asc) is a vital water-soluble nutrient that humans and other primates cannot synthesize, owing to mutations in the gene that produces L-gulono-γ-lactone oxidase (GLO), the essential enzyme catalyzing the final step of Vit-C formation. Therefore, humans must receive their Vit-C from dietary sources [1,2]. Mice, however, unlike primates, are able to produce their own Vit-C [3,4].

Vit-C has several pivotal physiological functions in the body, through acting as an electron donor. In addition to being a powerful antioxidant, it also helps to maintain vital tissue structures and functions, by protecting key macromolecules such as proteins, fats, and DNA from oxidation [4]. Vit-C also functions as an enzyme co-factor (co-activator) for a variety of the biosynthetic enzymes that are involved in the production of hormones and the production of metabolic energy [5], along with another category of regulatory enzymes for certain genes [6,7]. In a recent study, it has been shown that Vit-C plays a pivotal role in controlling gene transcription via its action on transcription factors and epigenetic modifying enzymes [8,9]. Moreover, the nutritional deficiency of Vit-C is frequently related to anemia, bleeding gums, infections, scurvy, capillary haemorrhage, delayed wound healing, atherosclerotic plaques, muscle degeneration, and neurotic disorders [7]. Vit-C is often supplemented in high amounts to make up for such deficiencies, although toxicity is uncommon when taken in high dosages, unlike for fat-soluble vitamins. Vit-C is used in the prevention and treatment of a broad spectrum of conditions, including diabetes [8], atherosclerosis [9], the common cold [10], cataracts [11], glaucoma [12], macular degeneration [13], stroke [14], heart disease [15], COVID-19 [16], and cancer. Recently, researchers have also begun to look into the function of Vit-C in the prevention and treatment of infection and immunity. Therefore, our review discusses the multiple functions of Vit-C, in terms of its anti-cancer functions, epigenetic regulation, and immunotherapy enhancement. We also provide a short overview of the current state of systems for scavenging ROS, especially in the context of their influencing high-dose Vit-C toxicity for the inhibition of cancer growth.

## 2. Historical Background and Justification of High-Dose Vitamin-C in Cancer Management

The notion that Vit-C may play a significant part in cancer prevention was originally postulated in the 1970s by Cameron and colleagues, who suggested that [17,18,19] high-dose Vit-C may increase the survival of cancer patients in their terminal stages. In the same decade, Pauling and Cameron conducted the first recorded research in which Vit-C was given to cancer patients. They demonstrated that giving 100 terminally sick cancer patients with 10 g of Vit-C daily led to excellent results, when compared to a thousand cancer patients who received the conventional treatment. It was shown that 10% of cancer patients who received Vit-C lived; while no malignancy cases that received the conventional treatment without Vit-C went on to survive [18]. The follow-up investigations supported these data. Murata and Morishige demonstrated, in research performed on Japanese patients with uterine malignancies and who received 5 to 30 g of Vit-C daily, that these individuals lived six times longer than those who received only 4 g of Vit-C daily. When a comparison was conducted between individuals who received Vit-C supplements and those who did not, it was shown that those who received Vit-C supplements had a 15% greater survival rate [20]. However, Moertel et al. found that high-dose Vit-C at 10 g per day had no advantage, when compared to placebo in two different investigations on 100 and 150 patients with late stage colorectal cancer (CRC) and patients with late stage cancers, respectively [21,22].

Now, new findings support the notion that high-dose consumption of Vit-C is associated with a decreased risk of the oral cavity, stomach, esophagus, pancreas, cervix, breast, and rectum cancers [23,24], and cancers with non-hormonal origins [25]. Furthermore, in terms of the amenable factors of risk for cancer, food counts as one of the most significant. Several independent study panels and committees have come to the conclusion that a high consumption of fruits and vegetables lowers the risk of developing various forms of cancer [26,27]; and in particular, it was shown that Vit-C intake was inversely linked to fatality rate [28]. In contrast, the consumption of vitamins A, C, and E, according to a research including 34,000 postmenopausal women, did not seem to be associated with a decreased risk for developing breast cancer [27]. In addition, according to some studies, intravenous Vit-C injection has been shown to be beneficial in the therapy of advanced malignancies [29,30]. 

Numerous mechanisms have been suggested to support the role of Vit-C in cancer treatment and prevention, including that of increasing the immune system activity; stimulating collagen synthesis; preventing metastasis, by inhibiting certain enzymatic reaction; inhibiting tumor-causing viruses; correcting for the lack of Vit-C, which is normally linked with cancer patients; wound healing in patients with cancer subsequent to surgery; improving chemotherapy sensitivity; decreasing chemotherapy toxicity; and neutralizing certain carcinogens [31]. Various recent experimental investigations have shown that when tumor cells are exposed to high doses of Vit-C, they suffer growth arrest [32,33]. Recent reports have also shown that Vit-C administration inhibits metastasis, tumor development, and inflammatory-associated cytokine production, while improving tumor inclusion and enhancing chemotherapy [34,35]. According to some reports, intravenous injection raises Vit-C levels over 70-fold when compared to oral delivery, and the efficacy of the therapy is inversely proportional to the excess amount of Vit-C [29,36]. For this reason, the ideal method of administration, the dosage, and the length of the treatment are being intensely debated. 

Recently reported pharmacokinetic data have improved our knowledge about Vit-C transport regulation and given more clues about the therapeutic effectiveness of Vit-C. This has increased the interest in reconsidering the possibility of utilizing Vit-C in the inhibition of cancer development. Despite the fact that the procedures in these reports vary, the majority of the current research on Vit-C and cancer explores the impact of high-dose Vit-C on the formation and development of cancers, as well as the mechanisms of action that govern the anti-tumor impact of Vit-C [37]. In addition, studies have refocused on the consequences and usefulness of high intravenous Vit-C doses in cancer treatment. In vitro, pharmacological doses of Vit-C from 0.3–20 mmol/L preferentially target and kill cancer cells, compared to the usual physiological levels of Vit-C, which are 0.1 mmol/L. This tumor-killing phenomenon is due to the pro-oxidant characteristics of Vit-C, which, at high concentrations, facilitates the formation of hydrogen peroxide (H_2_O_2_), which may be an agent responsible for the anti-tumor impact of Vit-C and its use as a pro-drug in cancer therapies [38,39]. However, determining the exact contribution of Vit-C to clinical results is challenging, because patients are usually receiving several therapeutic regimens at the same time [40]. As a consequence, although the therapeutic benefit of high-dose Vit-C in cancer remission or development has not been unambiguously established, intravenous (i.v.) administration of the vitamin in high doses can enhance quality of life, even in the advanced stages of the disease [40].

## 3. Different Oxidized Forms of Vitamin-C

Given that Vit-C can occur in several oxidative states, interestingly, ascorbic acid is oxidized by ROS or/and free radicals, resulting in the formation of a reactive anion intermediate radical (Asc^•−^), which is subsequently oxidized to de-hydroascorbic (DHA) acid [41,42]. DHA, which has a relatively short half-life of just a few minutes [43], is converted to around 1–5% of the Vit-C inside the human cells [44], and it can be transferred into the cell or hydrolyzed irreversibly into 2,3-Diketo-L-gulonic (2,3-DKG) acid (C_6_H_8_O_7_). When 2,3-DKG is broken down into Ethanedioic acid (C_2_H_2_O_4_) and (2R,3S)-2,3,4-Trihydroxybutanoic acid (C_4_H_8_O_5_), the Vit-C level is significantly decreased [45]. DHA is quickly converted to Vit-C within the cell, by interacting with reduced glutathione (GSH) [45,46,47]. NADPH then recycles the oxidized glutathione (glutathione disulfide (GSSG)) and converts it back into GSH [45].

## 4. Enzymatic Activities of Vitamin-C 

The biochemical activities of Vit-C may be ascribed to its chemical characteristics, which can donate electrons to a variety of molecules. Physiological ascorbate, which acts as an anti-oxidant at micromolar concentrations, may reduce ROS toxicity [42,48]. It may also act as a pro-oxidant at low plasma levels, which might be created via the i.v delivery of Vit-C [49]. Surprisingly, Vit-C also serves as a critical co-factor for many enzymes, easily giving electrons to prosthetic metal ions to attain a complete enzymatic action [44]. Overall, these proteins are divided into two categories: mono-oxygenases-containing copper and a-KG-, oxygen-, and iron-dependent dioxygenasesoxygen-, and iron-dependent dioxygenasesoxygen; and iron-dependent dioxygenases (α-KG; identified as 2-oxoglutarate (2OG))-dependent dioxygenases (α-KGDDs). α-KGDDs are iron-containing proteins that use oxygen (O_2_) as well as α-KG as an enzyme co-factor to produce succinate and CO_2_. α-KGDDs also catalyze various chemical processes involved in the construction of collagen, hypoxia-inducible factor 1-alpha (HIF1-α) stability, carnitine synthesis, tyrosine catabolism, and protein, DNA, and RNA de-methylation [50,51,52]. Accordingly, Vit-C is crucial for regulating a wide range of essential cellular activities.

## 5. ROS

As the therapeutic impact of high-dose Vit-C is strongly dependent on the formation of ROS, it is essential to define this term. The term “ROS” refers to a group of highly reactive chemical species formed when electrons escape from the mitochondrial electron transport chain (ETC; coenzyme Q) and interact with molecular O_2_, which is converted enzymatically to superoxide (O_2_^•−^) and dismutated to produce H_2_O_2_, which is then partially reduced to form hydroxide ions (OH^−^), hydroxyl radicals (HO^•^), and water (H_2_O) [53,54]. It is worth noting that the superoxide and the hydroxyl radical are together referred to as ROS free radicals [54]. Furthermore, a broad spectrum of substances may produce ROS exogenously. These include heavy metals, radiation, cigarette smoke, drugs, and xenosensors [55,56,57,58,59], among others. 

ROS play a key regulatory function in various metabolic pathways in living systems; hence, they are being continuously generated and eliminated. Under normal physiological circumstances, cells control ROS levels through their scavenging machinery [60]. However, under oxidative pressure, increased levels of ROS can result in DNA, protein, and lipid damage, which can potentially lead to cancer formation [61]. However, the action of ROS is essential for eradicating cancers at the molecular level. For these reasons, strategies to increase or eliminate ROS have been established. Tumors with higher ROS levels, for example, depend largely on their anti-oxidative stress defense systems. Drugs that elevate ROS can increase intercellular ROS amounts directly through ROS production (e.g., gadolinium, motexafin, elesclomol) or through inhibition of superoxide dusmutases (SODs) (e.g., ATN-224, 2-methoxyestradiol), and GSH (β-phenylethyl isothiocyanate (PEITC), buthionine sulfoximine (BSO)), which can produce massive ROS amounts, leading to cancer cell death [62,63]. However, under the low basal stress, normal cells appear to respond in a positive manner to enhanced ROS levels [63]. As a result, increasing ROS levels in all cells may be employed to selectively destroy cancer cells, a strategy discussed here in the context of high-dose Vit-C therapies.

## 6. Anti-Cancer Mechanisms of Vitamin-C

In recent years, numerous experimental reports have confirmed that low, millimolar range doses of pharmacological Vit-C may destroy tumors in vitro and prevent cancer development in vivo. The precise process by which certain tumor cells become susceptible to Vit-C, whereas wild type cells are unaffected is unclear. In fact, there is a wide range of mechanisms through which Vit-C may impact the progression of cancer. Conversely, the efficacy of this interaction is affected by a range of variables, such as the type of malignancy being managed and the different pathways that tumor cells utilize. In this section, we will look at how the anticancer mechanism of pharmacological ascorbate affects cancer cells.

First, cancer cells, owing to their defective mitochondria and increased metabolic reliance, are more sensitive to oxidative stress than normal body cells [64]. Oxidative stress may aid in tumor growth via ROS, by enhancing the cell development and increasing the genetic imbalance. However, an excess of ROS may destroy cancer, and to avoid this deleterious effect, cancer cells utilize a variety of mechanisms that may block the negative effects of ROS [42,65]. Since cancer cells can utilize ROS for their development and growth [66], anti-oxidant treatments have been investigated as potential anti-cancer strategies, based on the notion that ROS promotes tumor development. Various studies, on the other hand, have shown no convincing indication of the effectiveness of antioxidant therapy in inhibiting tumor growth [67]. On the contrary, the antioxidant treatments seemed to facilitate cancer development and cancer cells metastasis in animal models [68,69] and increase the possibility for the development of certain cancers in humans [70,71]. 

These outcomes indicate that various malignancies depend on anti-oxidants for survival and, as a result, pro-oxidant drugs may be amenable in this case. Undoubtedly, pro-oxidant anti-tumor treatments, such as radiation, have been used for the treatment of patients with cancer [72]. On the other hand, the existing anti-oxidant therapies often cause substantial collateral damage, making it difficult to use them regularly [72]. Therefore, high-dose Vit-C may avoid these issues by targeting multiple tumor hallmarks, while simultaneously demonstrating no toxicity.

### 6.1. High-Dose Vitamin-C Targeted the Intracellular Labile Fe(II) Iron Ion Hemostasis

**Figure 1:**
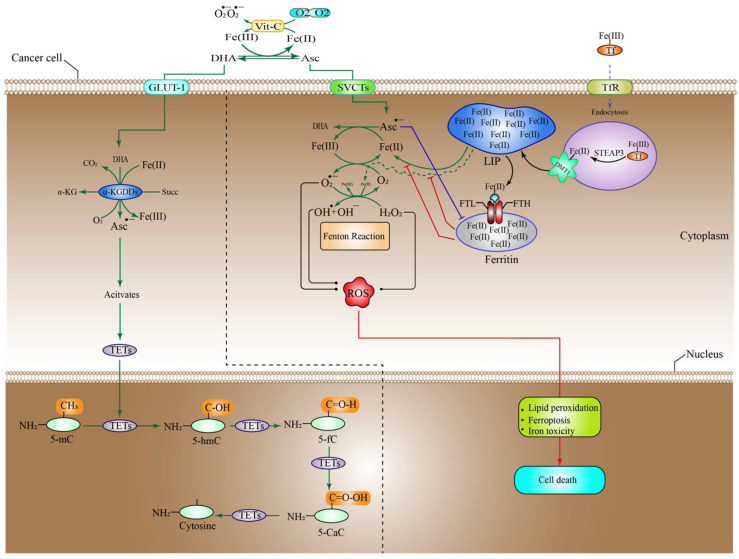
High-dose Vit-C targets the LIP imbalance and activates TET enzymes. First, the generated Asc^•−^ extracellularly enters the cancer cell through SVTCs, while at the same time Tf-Fe(III) complex enters the cancer cell via TfR1 by endocytosis. In the endosome, the captured Tf-Fe(III) undergoes acidification by STEAP3, and Fe(III) is reduced to Fe(II), which is subsequently transferred across the endosomal membrane by DMT1. Then, Fe(II) accumulates and generates the LIP that interacts with the Asc^•−^ and cellular O_2_ to produce DHA and Fe(III), which later produce H_2_O_2_, OH^•^, and O_2_^•−^ via the Fenton reaction and Haber–Weiss reaction, respectively [74,75]. The produced H_2_O_2_, OH^•^, and O_2_^•−^ are highly toxic to cancer cells, which can ultimately lead to lipid, protein, and DNA oxidation and may result in cancer cell death via apoptosis and ferroptosis. In addition, the amount of ROS generated via utilization of LIP can be inhibited by the FTH, which sequesters the LIP that is available to be used by Asc^•−^, which further inhibits ROS generation. In part, Asc^•−^ can inhibit FTH, and Fe-S results in higher amounts of LIP, and in turn in higher concentrations of ROS [77]. Second, intracellular ascorbate boosts TET enzymatic activity by influencing the DNA methylation, actively removing cytosine methylation via a cascade of oxidation reactions requiring O_2_, α-KG, Fe(II), and ascorbate via its activity as an α-KGDD. TETs first transform 5mC to 5hmC, then 5fC to 5- 5CaC. Next, by the base excision repair enzyme, TDG or replication-dependent dilution, 5fC and 5CaC are transformed into cytosine. Ascorbate ensures that TETs are continuously in a fully active state by recycling Fe(III) to Fe(II). See the text for agents working together with Vit-C to restore TETs function. Asc^•−^,Ascorbate radical; SVTCs, sodium–vitamin C cotransporters; Tf, transferrin; TfR1,transferrin receptor1, STEAP3, six transmembrane epithelial antigen of the prostate 3; DMT1, divalent metal transporter 1; LIP, labile iron pool; FTH, ferritin heavy chain; Fe-S, Iron(II) sulfide; α-KG, α-ketoglutarate; TETs, ten-eleven translocation enzymes; α-KGDD, α-KG-dependent dioxygenase; DHA, dehydroascorbic acid; 5mC, 5-methylcytosine; 5hmC, 5-hydroxymethyl cytosine; 5fC, 5-formylcytosine; TDG, thymine DNA glycosylase; 5CaC, 5-carboxylcytosine.

Iron (Fe) is a vital nutrient that the cells need as a co-factor to execute various processes, including metabolic activity, oxygen transport, and DNA synthesis [73]. Owing to its redox reactivity, iron can transport electrons, facilitate catalytic reactions, or bind to proteins and oxygen. Iron is found in the human body in the form of haemoglobin in red blood cells and growing erythroid cells. Although macrophages contain considerable quantities of iron, excess iron is retained in the liver cells [73]. Under normal conditions, iron is taken up by the majority of cells in the form of a transferrin (Tf)-Fe(III) complex that binds to the cell surface receptor transferrin receptor 1 (TfR1), mediating conformational change, after which it enters the cell through endocytosis [74]. In the endosome, after acidification, the endosomal six transmembrane epithelial antigen of the prostate 3 (STEAP3) reduces Fe(III) (ferric ion) to Fe(II) (ferrous ion), which is subsequently transferred across the endosomal membrane by divalent metal transporter 1 (DMT1). The cellular Fe(II) is now part of what is called the cellular labile iron pool (LIP), which is radially accessible for utilization in a variety of cellular processes [74]. However, LIP is toxic to the cells owing to the production of massive amounts of ROS. More specifically, an interaction between Fe(II) and H_2_O_2_ produces OH^−^ through the Fenton reaction. In addition, Fe(II) catalyzes the formation of OH^•^ and OH^−^ during the interaction between H_2_O_2_ and O_2_^•−^ (Haber–Weiss reaction) (Figure 1) [75]. Ascorbate can efficiently reduce free iron, thus recycling the cellular Fe(II)/Fe(III) to produce more OH^•^ from H_2_O_2_ than can be generated during the Fenton reaction, which ultimately leads to lipid, protein, and DNA oxidation [76,77,78]. It is worth noting that ascorbate recycling across the cell membrane might boost Vit-C-stimulated iron absorption from low-molecular-weight iron–citrate complexes. Ascorbate may also reduce cellular iron efflux [79]. These findings suggest that high-dose Vit-C may elevate cellular LIP concentrations, resulting in cellular death through ferroptosis, which is an iron-dependent type of controlled cell death, characterized by an excess of lipid peroxides on the cell membranes [80]. Similarly, the ferritin heavy chain (FTH) tightly controls and stores the cellular LIP [81], and this may be linked to the enormous loss of ROS that is generated by high-dose Vit-C. Interestingly, one study found that when high-dose ascorbate was combined with fasting mimicking diet (FMD), also known as short-term starvation (STS), it preferentially decreased FTH protein expression in *KRAS*-mutant cancer cells in vitro. These findings were further verified in vivo, where cycles of ascorbate+STS therapy substantially decreased FTH protein levels in cancer cells originating from HCT116 [77]. Additionally, ascorbate enhanced cancer cell LIP specifically by generating H_2_O_2_, which damaged Fe-S(iron sulfide)-containing proteins [82]. Although this seems to imply that ascorbate may cause LIP increases via disrupting proteins that make the ions inaccessible; thus, influencing the amount of ROS generated, additional research is required to corroborate this notion.

Various experiments have proven that Vit-C produces H_2_O_2_ extracellularly, which in turn inhibits tumor cells immediately [31,83,84,85]. However, the precise molecular processes behind this phenomenon are still unknown. Extracellular H_2_O_2_ may be produced spontaneously in the absence of iron by interacting with O_2_ when high-doses of Vit-C are introduced to the media [86,87]. Additional studies have shown that free irons, especially Fe(II), increase Vit-C autoxidation, leading to H_2_O_2_ production in cell-free and cell culture growth media [88,89]. Since Tf has been recognized to sequester most labile Fe(II) in vivo [89], these findings suggest that the capacity of ascorbate to generate H_2_O_2_ is an in vitro effect. 

**Figure 2:**
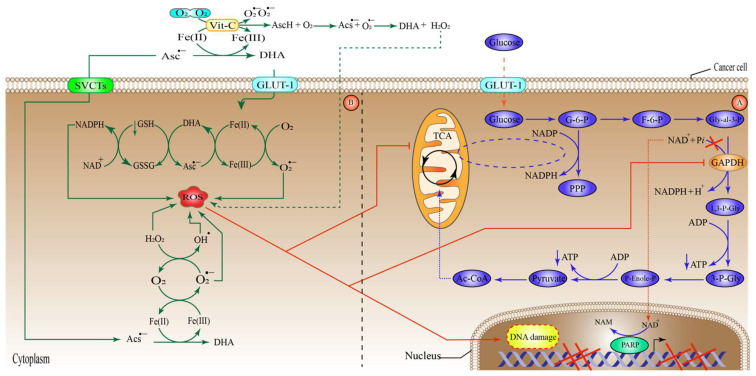
The anti-glycolytic and apoptotic effect of high-dose vitamin-C on tumor cell toxicity. High-dose Vit-C can destroy cancer cells by utilizing two different pathways that work in tandem. Initially, by producing hydroxyl radical (OH^•^) through the Fenton reaction, extracellular H_2_O_2_ may effectively kill cancer cells [102]. High amounts of Fe (III) ions in the cancer microenvironment promote the oxidation of Vit-C from the ascorbate radical to DHA and Fe (II). After the generation of Fe(II), the ion enters an oxidation process via O_2_, resulting in the formation of superoxide (O_2_^•−^), which will be consequently converted to H_2_O_2_ and O_2_ by superoxide dismutase (SOD). Transferrin in the extracellular space traps Fe (III) and binds to the TfR receptor, and oxidizes the Fe(III) to Fe(II), resulting in the formation of LIP inside the tumor cell. The generated extracellular H_2_O_2_ can also diffuse directly inside tumor cells via aquaporins [46], interacting with Fe(II) to form OH^•^, which is hazardous to cancer cells. Then, ascorbate and the ascorbate radical recycle Fe(III) to Fe(II), to form DHA, with the generation of intercellular ROS leading to cell death by apoptosis. Second, the extracellular oxidation process of ascorbate to DHA in large quantities results in DHA uptake by glucose transporter 1 (GLUT-1). DHA is then reduced by glutathione (GSH) and NADPH to high amounts of intercellular ROS, activating poly(ADP-ribose) polymerase (PARP), which in turn consumes cellular NAD^+^, a co-factor of GADPH. As a result of the inhibition of GADPH, ATP production via glycolysis stops, leading to energy crisis [46]. In addition, the intercellular ROS generated by DHA oxidation can raise the metabolites and enzymatic activity in the pentose phosphate pathway (PPP), block the tri-carboxylic acid (TCA) cycle, and increase oxygen uptake, disrupting intracellular metabolic balance and resulting in irreversible cell death, due to an energy crisis. G6P, glucose-6-phosphate; farctose-6-phosphate, F-6-P; Gly-al-3P, glyceraldehyde 3-phosphate; GSSG, glutathione disulfide; SVCT, sodium-dependent vitamin C transporters, ROS, reactive oxygen species; PPP, pentose phosphate pathway; 1,3-P-gly,1,3-phosphglycerate; 3-P-gly, 3-phosphglycerate; P-Enole-P, phospo enol pyruvate; Ac-Coa, acetyl coenzyme A; GADPH, glyceraldehyde 3-phosphate dehydrogenase.

In contrast to this notion, it was demonstrated that Asc^•−^ and H_2_O_2_ were generated in vivo upon i.v Vit-C administration of around 0.5 g/kg of body weight and that the generation was Vit-C-dose reliant (Figure 2) [90]. One study revealed that a daily intraperitoneal administration of high-dose Vit-C (4 g/kg) inhibited the growth of neuroblastoma and ovarian cancer in a xenograft model, with increased checkpoint kinase 2 (CHK2), as well as histone 2AX (H2AX) function in malignancies resulting in DNA damage, which may be an in vivo cancer effect. However, in this investigation, no link could be established between Vit-C and the in vivo generation of H_2_O_2_ [91,92]. The key question is whether extracellular H_2_O_2_ generated by pharmacological Vit-C contributes to the selective damage of cancer cells. For example, oxidation processes and/or reactive LIP in the medium or the serum in vivo may produce H_2_O_2_ in the extracellular space in both normal and cancer cells, in which case pharmacological Vit-C would have no therapeutic advantage over alternative pro-oxidant treatments. Cell respiration, DNA synthesis, the cell cycle, and epigenetics are all regulated by iron and haem-associated proteins [91,93]. As a result, tumor cells have a need for readily available Fe(II) to survive and proliferate. 

It is generally accepted that iron metabolism is altered in malignancies such as breast cancer, prostate cancer, and lymphoma via a different mechanism, involving an increase in the expression of various iron-intake pathways or the downregulation of iron exporter proteins and storage pathways [94]. For instance, the amount of Fe(II) ion in breast cancer cells is almost double that in normal breast tissues [95]. Furthermore, macrophages in the cancer microenvironment have been revealed to increase iron shedding [96,97]. Advanced breast tumor patients had substantially greater Fe(II) levels in their blood than the control groups without the disease [98]. Cancers with high amounts of iron might be more susceptible to mega-dose Vit-C than non-cancer cells, because they may produce higher amounts of H_2_O_2_ and OH^•^ via LIP. Other studies found that higher levels of ROS inside mitochondrial lung cells, glioblastoma, and gastric tumor cells increased the amount of LIP inside the cells through transferrin receptor (TfR); thus, enhancing the tumor cell susceptibility to Vit-C [78]. This correlation may explain why the co-delivery of Prussian blue and Vit-C managed to suppress the formation of peroxide and obliterate the anti-cancer activity of Vit-C [99]. A corresponding research found that cells of multiple myeloma had an increased LIP, due to their poor production of the iron exporter ferroportin 1, leading to selective susceptibility to Vit-C [100,101]. Despite these encouraging experimental results, researchers now need to investigate if there is a link connecting Vit-C susceptibility, ascorbic radical or H_2_O_2_ production, and cytosolic Fe (II) amounts in a variety of tumors and tissue samples. Evaluating possible biological markers or gene expression profiles to estimate Vit-C sensitivity, due to the higher LIP quantities, might also be beneficial (Figure 1).

### 6.2. High-Dose Vitamin-C Targeted Glycolysis and GLUT-1 

Cancer cells have a rapid rate of glucose metabolism, which leads to lactic acid fermentation, regardless of the presence of oxygen, a characteristic originally reported by Otto Warburg in the 1920s [103]. This is now called the Warburg effect, or metabolic reprogramming, which is required for cancer cells to survive and proliferate [104]. The Warburg effect is aided by *KRAS* or *BRAF* mutations, which increase the expression of SLC2A1 [46,47,105,106]. These findings imply that targeting cancer cells via the specific expression of GLUT-1 and the metabolic burden related to a greater dependence on glycolysis could be a feasible treatment approach. Indeed, high-dose ascorbate has been shown to attack these vulnerabilities in the CRC cells of the *KRAS* or *BRAF* [47]. Moreover, when Vit-C is supplied, it oxidizes to DHA, and then is readily transported by GLUT-1 in mutant cells of *KRAS* or *BRAF* competing with glucose [46]. DHA is quickly converted into ascorbate inside the cell by NADPH and GSH [46,107]. This decrease reduces the concentration of cytosolic antioxidants and raises the intracellular ROS amounts [46]

The increased ROS inactivates glyceraldehyde 3-phosphate dehydrogenase (GAPDH), via the oxidation of the active site in a cysteine residue. Furthermore, ROS activates poly (ADP-ribose) polymerase (PARP), which depletes NAD^+^ (a critical co-factor of GAPDH); thus, further reducing the GAPDH associated with a multifaceted metabolic rewiring. Hindering GAPDH can result in an “energy crisis”, due to the decrease in ATP production [46,108]. In support of this, high-dose Vit-C therapy generated substantial oxidative stress in breast cancer cell lines, resulting in ROS production followed by DNA damage, and depletion of key intracellular co-factors, such as NAD^+^. Furthermore, high-dose Vit-C recruited metabolites and increased the enzymatic activity in the pentose phosphate pathway (PPP), blocked the tri-carboxylic acid (TCA) cycle, and increased oxygen uptake, disrupting the intracellular metabolic balance and resulting in irreversible cell death, due to an energy crisis [109]. Another study found that high-dose Vit-C treatment exhibited a positive effect by increasing certain metabolites associated with the energy metabolism pathways upstream of glycolysis (PPP and citrate and cis-aconitate) [110]. However, high-dose Vit-C through generation of H_2_O_2_ decreased the metabolite levels downstream of glycolysis (NAD) and the TCA cycle, with the exception of citrate and cis-aconitate. Furthermore, NAD depletion impeded the glycolytic flow mediated by GAPDH between glyceraldehyde 3-phosphate (GAP) and D-glycerate 1,3-bisphosphate (1,3-BPG), resulting in reduced ATP generation and cancer cell death, through energy crisis [110]. Taken together, these outcomes imply that mega-dose Vit-C influences energy metabolism by producing tremendous amounts of H_2_O_2_.

Fully in line with the in vitro data, a daily i.v administration of Vit-C (4 g/kg) reduced cancer growth in *Apc*^−/−^; *Kras ^G12D^* mutant mice, but had no effect in *Apc*^−/−^ mice [46]. These results also indicate that ascorbate treatment may be applied to other malignancies exhibiting excess GLUT-1 production and high glycolytic activation. Recent investigations found that high-dose ascorbate preferentially eliminated gastric malignancies and von Hippel-Lindau (VHL) null renal tumors with an elevated SLC2A1 and glycolysis production [111,112].

Due to the fragile characteristics of DHA and the biological and chemical balance between DHA and ascorbate, determining the precise amount of DHA made by ascorbate is challenging. Regardless of this challenge, many studies have shown that, in vitro and in vivo, tumor cells with robust GLUT-1 rather than sodium-dependent Vit-C transporters preferentially transport ascorbate in the DHA isoform [41,113]. 

Although reports have anticipated that DHA is Vit-C’s most pharmacologically effective form, it is essential to demonstrate that Vit-C, instead of DHA, must be utilized in both preclinical and clinical anti-cancer treatments. Due to its great volatility at neutral pH [76], bolus therapy with mega-dose DHA has only transitory effects on tumor cells, both in vitro and in vivo. Furthermore, DHA breakdown produces a variety of unwanted compounds, including oxalate and 2,3-DKG, which may reduce the effectiveness of Vit-C treatment [76]. In cell culture, media, and the blood, on the other hand, ascorbate has a prolonged half-life. Since oxidative stress exists in the cancer microenvironment [114,115], large quantities of ascorbate would produce DHA rapidly and constantly. Furthermore, when millimolar quantities of ascorbate are oxidized to produce extracellular H_2_O_2_, as previously stated, large quantities of exogenous DHA are produced, as DHA is the primary oxidized form of Vit-C. Evidence in humans supports the impact of oxidative stress on tumor cells caused by high-dose Vit-C through the mechanism described above. Following high-dose i.v. pharmacological Vit-C treatment, individuals with glucose 6-phosphate dehydrogenase (G6PD) defects have been affected by hemolytic anemia [116,117,118,119]. 

Despite having high GLUT-1 production and relying on glucose for energy, similarly to mutant *BRAF* and/or *KRAS* cells, erythrocytes contain a more significant amount of antioxidative enzymes and exhibit an improved glucose transit into the PPP [120,121], to enhance NADPH production (Figure 2). This prevents erythrocytes from the effects of high-dose ascorbate in normal circumstances. In humans, red blood cells lacking G6PD cannot create sufficient NADPH, a necessary molecule for replenishing the low glutathione levels (GSH) generated by Vit-C-induced oxidative stress, resulting in erythrocyte loss and anemia [102]. As such, individuals who want to have an i.v. Vit-C therapy should have post-screening for a possible lack of G6PD performed, to tackle this issue. The notion that high-dose Vit-C’s fast oxidative stress effects arise in erythrocytes due to this hereditary condition highlights the significance of expanded GLUT-1 concentrations and glycolysis reliance on the oxidative damage stimulated by Vit-C.

### 6.3. High-Dose Vitamin-C Targeted the Hypoxic Enfironment 

The majority of solid cancers suffer from hypoxic pressure, because the tumor mass may constrain and block the nearby blood vessels, and cancer cells cannot invade new capillaries. To overcome this low oxygen availability, tumor cells utilize the transcription factor HIF1, which upregulates a wide variety of genes to promote their growth and survival [122,123,124,125]. HIF-1, is a hetero-dimeric transcription factor containing HIF1-α and HIF1-β [122,125]. The primary method by which O_2_ regulates HIF1-α function is via activation of asparagine hydroxylase (factor-inhibiting HIF (FIH)) and proline hydroxylase domain proteins (PHD1, PHD2, PHD3), which are referred together as hydroxylases HIF. PHDs meditate the hydroxylation of HIF1-α-containing proline residue in normoxic circumstances. 

**Figure 3:**
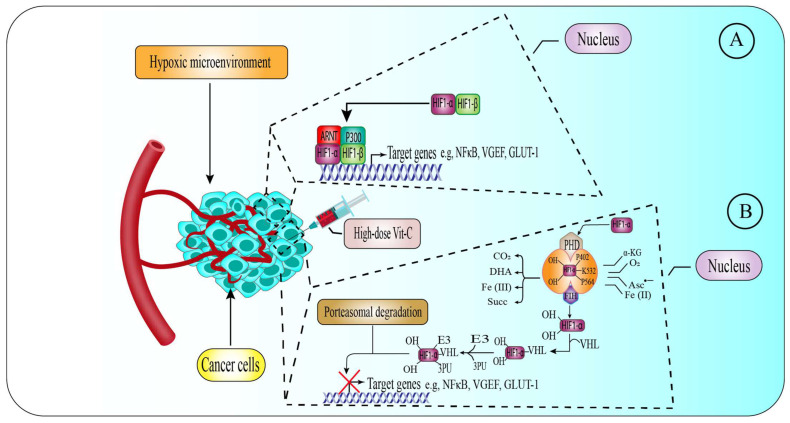
High-dose Vit-C targeting HIF1-α function. (A) In the tumor hypoxic microenvironment, hydroxylation is inhibited, and HIF-1α dimerizes with constitutively expressed HIF-1β, creating an active transcription factor HIF-1complex, which binds with p300 (a co-activator protein) and ARNT, together transcribing genes promoting angiogenesis, glycolytic metabolism, and survival. (B) During high-dose Vit-C administration, HIF hydroxylases, asparagine hydroxylase HIF, and PHDs utilizing ascorbate as a critical cofactor for their optimal activity, these proteins belong to the large family of the α-ketoglutarate (α-KG)-dependent dioxygenase (αKGDD). When the ascorbate availability is high, with sufficient O_2_ and Fe(II), HIF inhibits the function of HIF1-α.; consequently, HIF1-α is hydroxylated at proline residues by PHDs, followed by the binding of VHL (tumor suppressor protein), which attracts an E3-ubiquitin ligase enzyme that marks HIF1-α for degradation by proteasome and results in reduction of HIF1-α subunits inside the cancer cell. FIH then mediates the hydroxylation of an asparagine residue, N806, on HIF1-α. This hydroxylation stops p300 from combining with the HIF complex; this inhibits the HIF-1 transcription activity, resulting in downregulation of multiple downstream genes important in cancer development and growth [125,126,127,128,129,130,131]. HIF, hypoxia-inducible factor; FIH, factor-inhibiting HIF; ARNT, Aryl Hydrocarbon Receptor Nuclear Translocator; PHDs, proline hydroxylase domain proteins; VHL, von Hippel-Lindau; Succ, succinate.

The VHL cancerous suppressor protein binds to the prolyl-hydroxylated HIF1-α, which activates an E3-ubiquitin ligase, targeting it for degradation by the proteasome. In contrast, FIHs hydroxylate specific asparagine residues on HIF1-α, blocking them from binding with the p300 transcription factor and, therefore, reducing the HIF-1 transcription function. Together, HIF hydroxylases are Fe(II)-containing α-KGDDs that require α-KG and O_2_ for proper action. PHDs and FIHs are inactive in hypoxic environments, due to their reduced affinity for O_2_ compared to other KGDDs [125,126,127,128,129,130,131], resulting in HIF-1 maintenance and activation. To recycle Fe(II), HIF hydroxylases need Vit-C as a co-activator. As a result, ascorbate-deficient cells may have an enhanced HIF1-α function, possibly leading to tumor development. Ascorbate therapy may increase the activity of HIF hydroxylases, decreasing HIF1-α action and reducing tumor development (Figure 3) [132,133]. 

Vit-C has been shown to suppress HIF1-α-dependent tumor growth [134,135]. Vit-C levels were also shown to be negatively linked to HIF1-α production in thyroid abrasions [136], and an in vitro investigation found that Vit-C therapy reduced HIF1-α and GLUT-1 production in thyroid tumors [136]. In experiments with Gulo^−/−^ lab mice with lung cancer, tumors grew slowly when high-dose Vit-C was administered in drinking water (3.3 g/L) or injected daily at 1000 mg, particularly in comparison to Gulo^−/−^mice given low-dose (0.33 g/L) Vit-C in drinking water [137,138,139]. Here, cancers treated with high Vit-C also had lower levels of HIF-α and VEGF, and significantly decreased micro-vessel volume, when compared with mice in the control group. Furthermore, previously conducted research indicates a link between Vit-C, HIF-1 activation, and cancer development. Utilizing tissues from subjects with renal cell cancer (RCC) and CRC, researchers discovered that cancers with the most potent HIF1 function were those lacking Vit-C in the tumor microenvironment (TME) [140,141,142,143]. Individuals with CRC who had high Vit-C levels in their tumors also had a positive patient outcome and a relatively long post-surgery survival [140]. As a result, the findings indicate that high-dose Vit-C therapy may inhibit tumor development via controlling HIF-α. However, all investigations, so far, have shown correlation rather than causation. HIF-1 may be stabilized, even in normoxic circumstances, as seen in RCC; and destruction of the VHL tumor suppressor blocks HIF-α-degradation in normoxic conditions. In contrast to isogenic VHL-proficient cells, RCC cells lack VHL after receiving Vit-C in the same conditions [112]. Interestingly, the higher levels of GLUT-1 in cells lacking VHL under normoxic conditions enhanced DHA absorption, resulting in greater ROS formation and cell death compared to VHL-competent cells. In line with this, Glut-1 knockdown conferred tolerance to Vit-C toxicity in RCC cells lacking VHL. In addition to VHL mutations, alterations in two cancer suppressor proteins in the TCA, fumarate dehydrogenase (FH) and succinate dehydrogenase (SDH), may enhance HIF1-α stability and activity. SDH and FH loss-of-function mutations result in a buildup of succinate and fumarate, respectively [144]. As a result, the higher amounts of succinate and fumarate produced by these gene alterations may compete with α-KG, block the action of HIF hydroxylases, and therefore promote normoxic HIF1-α activity in vitro [131,145,146]. Since, SDH and FH mutations are related to the progression of many cancers, including paraganglioma, RCC, pheochromocytoma, and endocrinecancers [147,148,149,150], investigation of the Vit-C therapy warranted for these mutant cells resulted in suppression of HIF activity and decreased tumor growth and development in vivo studies. 

### 6.4. High-Dose Vitamin-C Targeted NF-κB in the Tumor Microenvironment

The nuclear factor kappa-light-chain-enhancer of activated B cells (NF-κB) orchestrates several genes responsible for signaling processes involved in inflammation, immune response, cell proliferation, differentiation, and survival, through a large group of proteins [151], containing NF-κB1 (p50), NF κB2 (p52), RelA (p65), RelB, and cRel, whih together mediate the expression and activation of target genes by binding as hetero- or homo-dimers to a specific DNA component, known as the κB enhancer [152]. The proteins of the NF-κB family are typically sequestered in the cellular cytoplasm by a category of suppressive proteins, which include IκB (IκBα, IκBβ, IκBγ, IκBε, Bcl-3, p100, and p105) family members and associated proteins containing ankyrin repeats. These proteins mediate the attachment of NF-κB to the transcription factors’ DNA binding sequences, rendering them transcriptionally inert [153,154]. The most important family member of the IκB is the IκBα [155], and its phosphorylation through IκB kinase (IKK) complex enhances the activation of the NF-κB [156]. Not surprisingly, NF-κB has been implicated in cancer cell development, as well as in genetic and epigenetic alterations, cancer stem cell development, cellular metabolism, and therapeutic resistance in TME [157,158,159,160,161]. Several studies have shown that Vit-C administration could result in the suppression of the NF-κB signaling pathways at one or more stages of their activation process. The first study to propose that Vit-C can inhibit NF-κB was reported by Bowie et al. [162]. They utilized high-dose Vit-C (5–20 mM) and discovered that inhibition was achieved by inhibiting NF-κB release from IκB, rather than by restricting the protein from binding to DNA. Surprisingly, in ECV304 and HUVEC cell lines, Vit-C (20 mM) inhibited TNF-α- and IL-1β-induced IKK phosphorylation of the IκΒα pathway and the subsequent binding of the NF-κB to DNA enhancer. Furthermore, Vit-C increased p38 mitogen-activated protein kinase (MAPK) activity via the indirect negative feedback that interferes with the IKK and the TNF-α receptor [163]. In line with this, another study demonstrated that TNF-α-induced NF-κB activation was inhibited by DHA through a direct effect on IKK-β kinase activity, which was fully independent of p38-MAPK [164]. Here, it should be noted that, the action of Vit-C-mediated NF-κB inhibition was not through ROS induced mechanisms. 

**Figure 4:**
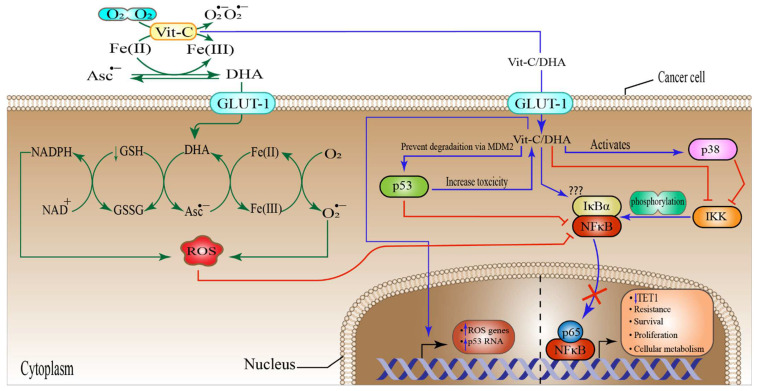
High-dose Vit-C targeting the NF-κB. High-dose Vit-C can generate massive amounts of ROS through the Fenton reaction and Haber–Weiss reaction by recycling Cu(II)/Cu(I) and Fe(II)/Fe(III), which in turn inhibits the NF-κB. Vit-C can also increase the expression of genes that produce p53 and ROS, in addition to activating and protecting p53 from degradation by MDM2, after which the activated p53 can inhibit the NF-κB and increase Vit-C toxicity. Vit-C can directly inhibit IKK and activate p38, which in turn blocks IKK, which releases NF-κB from IκBα by phosphorylation. If the NF-κB is successfully activated, it can initiate cancer growth and development via various pathways. Vit-C, vitamin C; MDM2, Mouse double minute 2; ROS, reactive oxygen species; IKK, IκB kinase.

Similarly, a very recent study found that Vit-C and DHA suppressed the LPS-activated IKK/IκB/NF-κB pathway, by inhibiting IKKα/β and IκBα phosphorylation. However, DHA suppressed the translocation of the IKKα/β more effectively than Vit-C [165]. Moreover, through a direct effect, the transition between Vit-C and DHA mediated by Cu(II)/Cu(I) recycle resulted in massive ROS generation, which in turn inhibited the inflammation mediated by NF-κB. This impact was explained by the fact that low levels of ROS activate the IKK/IκB/NF-κB signaling pathway, but high levels of ROS inhibit it by altering different cysteine residues in IKK [165]. Although most of these studies showed a rather indirect suppressive effect of Vit-C on NF-κB activation, one study demonstrated that Vit-C could directly inhibit NF-κB via ROS generation [165]. These noteworthy discoveries paved the way for the use of Vit-C in malignancies with abnormal NF-κB expression, which is a key factor in tumor growth and development. For instance, it has been shown that Vit-C at a very low concentration (5 µM), in combination with methotrexate (MTX), resulted in the activation of p38 in triple negative breast cancer (TNBC) [166]; and this, in turn, could further inhibit the NF-κB pathway. Moreover, treatment of the esophageal cancer (SKGT-4) cell line with 5-Fu (10 µM) and cisplatin (10 µM) enhanced the nuclear translocation of p65; however, Vit-C pretreatment (20 mM) suppressed this translocation. Interestingly, pretreatment with Vit-C had no effect on IκBα levels [167]. Again, this inhibition was not due to ROS generation. In addition, there is the evidence that overexpression of p65/NF-κB suppresses the TET1 expression in triple-negative breast cancer, skin cutaneous melanoma (SKCM), thyroid carcinoma (THCA), and lung adenocarcinoma (LUAD) cell lines [157]. This repression was directly caused by the attachment of the p65 to the TET1 promoter region. Arguably, high-dose Vit-C treatment may inhibit p65/NF-κB attachment and, at the same time, restore TET1 activity, as mentioned previously. Furthermore, several studies suggested that Vit-C could enhance p53 expression, which is a known inhibitor of NF-κB [168]. One study on isogenic cancer cells reported that overexpression of p53 increased ascorbate (5–10 mM) cytotoxicity dramatically, by 43%. Additionally, p53 expression elevated ascorbate-induced H_2_O_2_ generation, whereas ascorbate also increased p53 and ROS gene mRNA levels [169]. Moreover, ascorbate protected against p53 degradation via mouse double minute 2 (MDM2) ubiquitination [169]. Similar results were also found in cervical cancer and CRC cell lines, in which case combined treatments of 10–100 µM cisplatin + 100 µg Vit-C markedly raised p53 expression and strongly induced cellular ROS generation [170,171]. In line with this, Hahm et al. [172] utilized B16F10 murine melanoma cells and reported similar observations. In 2001, Reddy and colleagues demonstrated that Vit-C (1µM) upregulates and stabilizes the p53 gene involved in cancer control (Figure 4) [173]. Collectively, these findings point ay the possibility that the interaction between high-dose ascorbate and p53 might suppress NF-κB in a synergistic manner. Interestingly, inactivation of the NF-κB has been reported to induce ROS and increase intracellular LIP in cutaneous T-cell lymphoma (CTCL), followed by downregulation of FTH [174]. These results suggest that high-dose Vit-C might enhance the production of massive amounts of ROS and intracellular iron, by inhibiting NF-κB, which may increase the possibility of cancer cell death by apoptosis and ferroptosis. 

### 6.5. High-Dose Vitamin-C Implies Epigenetic Regulation in Tumor Microenvironment

Epigenetic alterations are a hallmark during malignancies [175]. These, in turn, affect several critical genes that have pivotal roles in epigenetic control, including the ten-eleven translocations (TET 1,2,3) and JMJC families, which are often altered or transcriptionally downregulated in hematological [176,177] and solid malignancies [178]. JMJC mutations are substantially less prevalent, with variations in KDM5A and KDM6A being detected in only approximately 1% of cases [132]. 

Interestingly, 5-hydroxymethylcytosine (5hmC) level reduction is associated with many malignancies and may have a poor prognostic effect [179]. Consequently, TETs mutations can lower the 5hmC levels and are thought to be associated with a lack of substrates and co-factors for the optimal TET activities, such as Vit-C and O_2_ in the hypoxic tumor TME. The TET enzymes catalyze the 5-mC to 5-hmC, 5-formylcytosine (5fC), and 5-carboxylcytosine (5caC). These oxidized mCs are the primary intermediates in DNA de-methylation through base-excision repair (BER) or replication-dependent dilution [178]. TET2 is frequently altered or deleted in the germ line cells of myeloid and lymphoid cancers [180]. TET2 was shown to acquire gain-of-function genetic alterations, especially in iso-citrate dehydrogenase 1 and 2 (IDH1 IDH2) in acute myelogenous leukemia (AML) [181]. IDH1 and IDH2 are proteins that convert iso-citrate to α-KG in the mitochondria and cytosol. A neomorphic genetic mutation in IDH1 or IDH2 alters their function. This results in the accumulation of onco-metabolite 2-hydroxyglutarate (2-HG); thus, inhibiting the activity of α-KG-dependent proteins such as TET2, reducing 5hmC, boosting DNA methylation, and eventually positively modifying gene expression to affect cancer growth [182]. Specifically, Vit-C as a co-factor transfers an electron from Fe(II) to Fe(III), which ultimately activates TET proteins (Figure 1).

Notably, an in vitro study found that treating peripheral T-cell (PTCL) lymphoma and large B-cell (DLBCL) cells with high-dose Vit-C (1–10 mM) enhanced TET activity, which improved DNA demethylation. However, this epigenetic alteration was not due to H_2_O_2_. Furthermore, Vit-C therapy enhanced the expression of SMAD1, a tumor suppressor gene known to be inhibited by methylation and associated with greater lymphoma cell chemo-sensitivity [183]. In the same context, another study reported that a high Vit-C dose (1 g/kg), in the treatment of clear-cell renal cell carcinoma (ccRCC) cells, diminished methylation and recovered the 5hmC levels through TET stimulation [184]. In support of this, a pharmacologic dose of Vit-C resulted in increased intratumoral 5hmC levels and inhibited ccRCC development in vitro and in vivo. Moreover, administration of Vit-C (1 mM) resembled TET2 recovery, by increasing 5hmC production in Tet2-deficient mice primary murine hematopoietic stem and progenitor cells (HSPCs), as well as suppressing human leukemic colony production and leukemia development in human primary leukemia [185]. 

In addition, Vit-C promoted DNA hypomethylation and increased TET2-dependent gene expression in human leukemia cell lines. In addition, TET-mediated the DNA oxidation after Vit-C therapy, also making leukemia cells more susceptible to PARP inhibition. Furthermore, Tet2 restoration elicited a p53 transcriptional signature, indicating a DNA damage response. Moreover, upregulation of PARP, DNA-damage-producible gene (GADD45), and DNA glycosylases were associated with DNA demethylation during Tet2 restoration [185]. Significantly, a considerable number of subsequent studies have backed up this notion. For example, a daily regime of high-dose Vit-C (4 g/kg) recovered TET2 activity in TET2-deleted animal mice with leukemia, resulting in increased DNA demethylation and the synthesis of the proteins necessary for the growth of myeloid cells [185]. Remarkably, in pancreatic ductal adenocarcinoma (PDAC) cell lines, global 5hmC content was significantly raised following treatment with Vit-C (2 mM), and this was attributable to an increase in TET2 expression.

Intriguingly, Vit-C has been shown to raise 5hmC levels in the promoter region of the PH domain leucine-rich repeat protein phosphatase 2 (PHLPP2) tumor suppressor gene and boost PHLPP2 expression. Vit-C partially overcame the inhibition of pancreatic cancer cells when PHLPP2 expression levels were downregulated. These findings revealed the unique and specific mechanism of epigenetic changes that underpin Vit-C inhibition in PDAC [186], and suggested that PHLPP2 may be a potential biomarker and epigenetic candidate for Vit-C therapy in PDAC.

Meanwhile, another study discovered that in vitro Vit-C therapy augmented DNA demethylation promoters at enhancers of hematopoietic cell development-related zones and improved the expression of several critical genes in murine progenitor cells with mutation in IDH1 [187]. Furthermore, according to Gerecke et al., the combination of ML309 (10 μM) and Vit-C (1 mM) inhibited the mutant colon cancer cell lines harboring the IDH1 allele with an elevated content of 2-hydroxyglutarate ((2-HG), the onco-metabolite) and induced a noticeable decrease in 2-HG, bringing it back to levels equivalent to those in wild type cells. This reduction was accompanied by increased global DNA hydroxymethylation and enhanced gene expression of specific tumor suppressors (BAD, p16, 14-3-3 protein). It also directly affected the mRNA expression of epigenetically controlled genes (TET1) [188]. Here, it should be noted that most of these investigations utilized catalase (CAT) in the media, to overcome the oxidative stress produced by high-dose Vit-C. Moreover, to prevent the changes in intracellular ROS levels, 2-phosphate l- ascorbic acid was used; a derivative of Vit-C that is stable and not affected by oxidization in normal culture media. 

Vit-C appeared to increase 5hmC levels for blood malignancies and reduce the metastatic tumor development of melanoma and bladder cancers [189,190], suggesting that the Vit-C therapy may be effective for solid tumors with low 5hmC levels or low levels of TET expression.

Mechanistically, in a randomized, placebo-controlled clinical trial of myelodysplastic syndrome patients, oral administration of dose Vit-C (500 mg) and 5-azacytidine (100 mg) significantly increased the global 5hmC/5mC levels compared to placebo [191]. Coincidentally, low production of SLC2A3 in AML is accompanied by a poor response to Vit-C uptake, which directly affects the restoration of the TET2 enzyme function. As a result, in order for Vit-C to restore TET2 activity, significant levels of SLC2A3 expression are required [192]; suggesting that SLC2A3 might be a successful biomarker to predict how well Vit-C therapy might work in AML. 

Consistently, Vit C (200 µM) significantly improved the antileukemic activity of the histone methylation inhibitor 3-deazaneplanocin-A (DZNep) (0.5 µM) and the DNA methylation inhibitor 5-aza-20-deoxycytidine (5-Aza-CdR) (0.5 µM) via EZH2. This finding shows that histone methylation and DNA methylation work together to suppress gene expression. This relationship is essential in leukemogenesis, and it offers a strong case for using epigenetic drugs to reverse the gene silencing caused by DNA and histone methylation in AML treatment [193]. Indeed, these promising results need to be validated using high-dose Vit-C in animal models. Similarly, a recent study discovered that high-dose Vit-C inhibits the development of ccRCC cells in vitro by activating TET2 [194], implying that TET2 may be a potential therapeutic target for the clinical treatment of ccRCC patients.

Taken together, epigenetic regulatory enzymes and agents play a crucial role as tumor suppressors. The capacity of high-dose Vit-C to promote 5hmC production and DNA hypomethylation is of considerable importance in the preservation of genomic integrity, and in cancer prevention and therapy. 

## 7. High-Dose Vitamin-C Enhances Cancer Immunotherapy

Numerous tumors resist the immune response by expressing high amounts of checkpoint proteins, such as programmed cell death 1 (PD-1) and cytotoxic T lymphocyte antigen 4 (CTLA-4) [195]. Therefore, anti-checkpoint (ICP) medicines targeting PD-1 or programmed cell death ligand 1 (PD-L1) and CTLA-4 have been licensed for the treatment of a variety of cancers. When used as monotherapy regimens, these medicines significantly increase survival rates and are relatively safe [196]. However, treatment failure occurs in more than half of individuals treated with these medicines as monotherapies. CTLA-4 and PD-1 combined inhibition has now been suggested to improve patient response rates and survival rates [196]. However, 50% of patients were subject to a higher toxicity as a result of the therapy regimen [197]. To reduce the toxicity and maximize the efficacy of these combinations, they must be used with drugs that are efficient in activating immune cells, namely cytotoxic T cells (CTLs), natural killer cells (NK cells), and antigen-presenting cells (APCs). 

In this regard, high-dose Vit-C has been proven to enhance cancer immunotherapies in several in vivo and in vitro studies. Accordingly, a combination of high-dose Vit-C (4 g/kg) with anti-CTLA-4 (200 μg) and anti-PD-1 (250 μg) antibodies resulted in significant tumor impairment and remission via the infiltration and activation of anti-cancer adaptive immunity (CD8 T and CD4 T cells), especially CD8 T cells. This activation was revealed by the production of higher interferon-gamma (IFN-γ) and the increased effectiveness of immune checkpoint inhibitors in various animals and in vitro models [35]. Along the same line, after addition of high-dose Vit-C (1.5 M) with anti-PD1 (200 μg) antibodies in a lymphoma mouse model, high-dose Vit-C therapy improved tumor immune recognition, and resulted in enhanced macrophage and cytotoxic T cell infiltration, which is not seen with anti-PD1 treatment alone. In addition, anti-PD1 antibodies inhibited the PD-1/PD-L1 axis’s inhibitory effect on APCs, CD8^+^ T, and NK cells. However, anti-PD1 alone is more effective in directly activating these cells than high-dose Vit-C. Indeed, the combined therapy significantly increased the production of IL-12 by APCs and granzyme B by cytotoxic cells (CD8^+^ T cells and NK cells) as compared to either of the drugs alone [198]. An additional study in the RCC mice model demonstrated that high-dose Vit-C therapy alone led to tumor infiltration by both CD4+ and CD8^+^ T cells and elevated the ratio CD8^+^/CD4^+^, while regulating the production of numerous cytokines and chemokines. Anti-PD-L1 antibody injection alone increased intratumoral CD4^+^ and CD8^+^ T cells significantly. However, the infiltration of CD4^+^ and CD8^+^ T cells, as well as the CD8^+^/CD4^+^ ratio, increased considerably when high doses of Vit-C were coupled with anti-PD-L1 antibodies. Specifically, Vit-C stimulates TET2, which in turn activates IRF1, which eventually binds to STAT1, to enhance the expression of PD-L, making tumor cells more susceptible to immunotherapy [199]. These findings indicate that high-dose Vit-C and the anti-PD-L1 antibody can act synergistically to eliminate tumor cells, and that the immunotherapy effect of Vit-C-anti-PD-L1 combination is TET2-dependent.

Supporting this notion, in mouse melanoma and colon tumors, high-dose Vit-C dramatically enhanced T helper 1 (Th1) chemokines and tumor-infiltrating lymphocytes in the IFN-γ/JAK/STAT/TET axis, resulting in improved anti-tumor immunity and anti-PD-L1 effectiveness. Specifically, Vit-C enhanced IFN-γ-stimulated production of three Th1-type chemokines and PD-L1 genes in a TET2-dependent sense, where the activated TET2 binds to and increases the levels of 5hmC in the CXCL10 and PD-L1 promoters. However, loss of TET2 significantly decreased the ability of Vit-C to activate tumor-infiltrating CD8^+^ and CD3^+^ cells, indicating that TET2 is the primary target for Vit-C in enhancing the effectiveness of the anti-PD-L1 therapy [200]. TET activity may be used as a biomarker to predict the efficacy and response of patients to an anti-PD-1/PD-L1 therapy, and high-dose Vit-C could be regarded as an adjuvant to immunotherapy through the achieved stimulation of the TET activity, especially for solid tumors expressing significantly lower levels of 5hmC.

**Figure 5:**
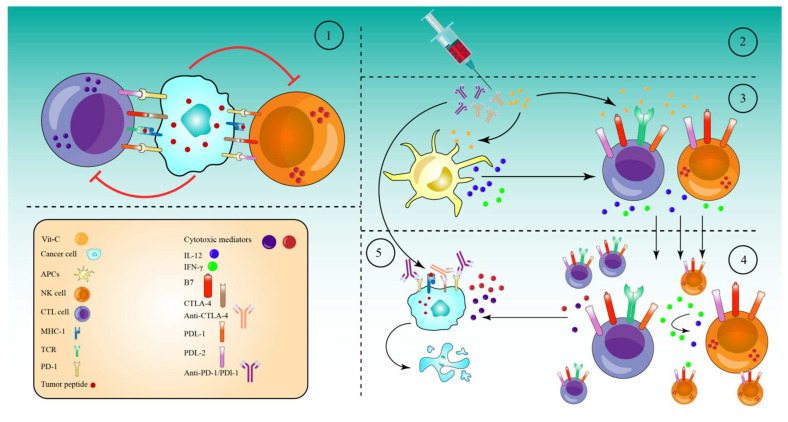
Systematic effect of high-dose Vit-C on cancer immunotherapy. (1) Cancer cells resist and inhibit the immune response to prevent successful cancer eradication. (2) High-dose Vit-C combined with ICP therapy. (3) High-dose Vit-C activates APCs to increase cytokines (IL-12 and IFN-γ), and also enhances the phagocytic activity of the APCs; in addition, high-dose Vit-C can also activate and increase the intratumoral infiltration of both CTLs and NK cells. (4) As a result, these cells in turn produce more cytokines, chemokine, and cytotoxic mediators (perforin and garnzyme). (5) The ICP therapies bind to their target proteins on cancer cells and make them susceptible to potential cellular lysis mediated by CTLs and NK. ICP, immune check point; Vit-C, vitamin-C; APCs, antigen presenting cells; IFN-γ, interferon gamma; IL-12, interleukin 12; NK cells, natural killer cells; CTLs, cytotoxic T lymphocytes.

Based on the ability of high-dose Vit-C to produce massive amounts of ROS in tumor cells, a recent study reported that Vit-C and oncolytic adenoviruse (oAds) combination therapy increased the production of ROS significantly, as compared to Vit-C or oAds monotherapies. This increased production of ROS, in turn, induced immunogenic cell death (ICD), which was confirmed by the increase in the calreticulin (CRT) translocation onto the cell membrane and by the upregulation of two ICD markers, heat shock protein 90 (HSP90) and high mobility group box 1 (HMGB1), which successfully stimulated dendritic cell (DC) maturation [201]. The results of the combined treatment were further evaluated in a variety of tumor-bearing mice. As a result of DC (CD11c^+^MHC-II^+^, CD80^+^, and CD86^+^) maturation and activation, the Vit-C and oAds recipe also expanded the numbers of CD3^+^, CD4^+^, and CD8^+^ T cells, particularly CD8^+^ T cells (IFN-γ, STAT1, CD137, IL-12p35, Granzyme B, and Perforin), in the tumor tissues and caused the upregulation of genes associated with T-cell motility, for example CCL3, CCL4, CCL5, and CXCL10 [201]. In addition, the combination therapy also significantly reduced the proportion of Regulatory T cells (Tregs-FoxP3^+^CD25^+^) and increased the ratios of CD4^+^ T cells to Tregs and CD8^+^ T cells to Tregs. At the same time, neither Vit-C or oAds single-therapy nor Vit-C and oAds combination therapy diminished the ratio of MDSCs (Gr-1^+^CD11b^+^) in cancer tissues. However, single-therapy with Vit-C alone or oAds alone reduced the ratio of Tumor-associated macrophages (TAMs-F4/80^+^CD11b^+^) in tumor tissues. While the combined Vit-C and oAds reduced the proportion of TAMs (M2 polarized TAMs (CD206^+^ F4/80^+^CD11b^+^) in tumor tissues. All these events, together, mediated the tumor suppression in tumor-bearing mice and in cell culture treated with Vit-C and oAds combined therapy [201] (Figure 5).

Altogether, these findings imply that high-dose Vit-C and ICP therapies have synergistic anti-tumor effects. 

It should be noted that, to the best of our knowledge, only a few studies have reported the utilization of high-dose Vit-C and immunotherapies to date. Indeed, given the huge promise of immunotherapy in anti-cancer treatment, these results are very encouraging, and they indicate a possible combination approach for transforming the tumor microenvironment and increasing the therapeutic breadth of immunotherapy.

However, no clinical trials have been conducted on the use of Vit-C in conjunction with ICB treatments, and additional investigations are required to give more emphasis on this important interaction; paving the way towards effective clinical trials.

## 8. Antioxidant Systems That Might Inhibit the Effect of High-Dose Vitamin-C Therapy

**Figure 6:**
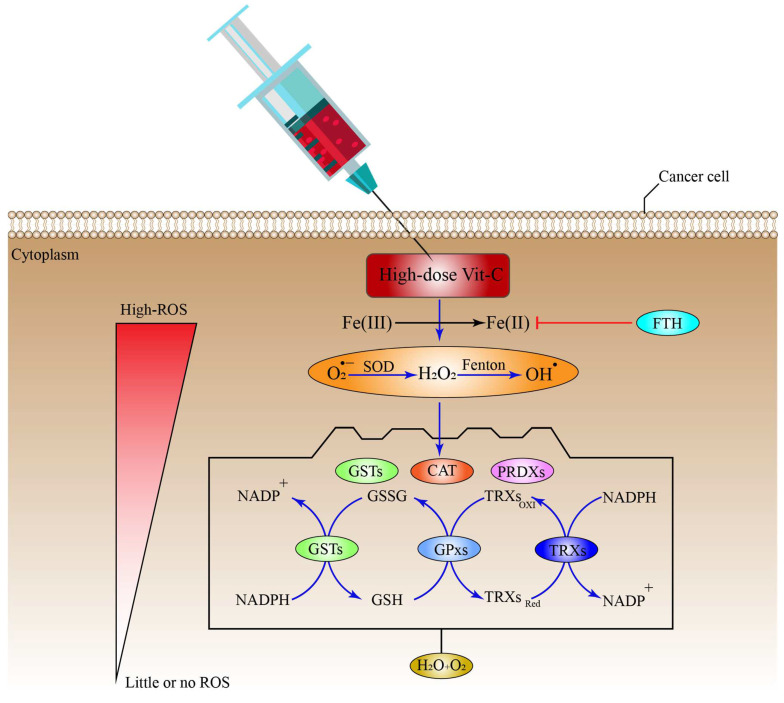
Inhibition of ROS generated by high-dose Vit-C via different antioxidant systems. Cancer cells may express different antioxidant systems that may together scavenge ROS. Through recycling of Fe(II)/Fe(III) and the Fenton reaction, high-dose Vit-C produces massive ROS concentrations that, in turn, damage cancer cells. In this regard, SOD converts O_2_^•−^ to H_2_O_2_, then CAT, PRDXs, GSH, TRXs, GSTs, and GPxs, together, convert the produced H_2_O_2_ to H_2_O and cellular O_2_, and this may prevent the production of OH^•^. In addition, FTH prevents the formation of ROS through massive sequestration of the LIP. Moreover, this collectively inhibits ROS generation. Vit-C, vitamin-C; SODs, superoxide dismutases; CAT, catalase; PRDXs, Peroxiredoxins; GSTs, glutathione-S-transferases; TRXs, Thioredoxins; GPxs, glutathione peroxidases; GSH, reduced glutathione; GSSG, glutathione disulfide; FTH, ferritin heavy chain; LIP, labile iron pool.

Cells use a range of mechanisms to counteract ROS-induced oxidative stress, all of which are mediated by different antioxidant regulatory systems, including CAT, superoxide dismutases (SODs), glutathione peroxidases (GPXs), and Peroxiredoxins (PRDXs) [63,202,203]. Furthermore, these systems convert O_2_^•−^, OH^•^, and H_2_O_2_ into H_2_O and O_2_ molecules. Additional systems that control ROS include co-factors for the PRDX and GPX-catalyzed processes of reduced GSH and reduced thioredoxin (TRX), respectively; while, GSH is also employed by glutathione-S-transferases (GSTs) to inhibit ROS production (Figure 6), (Table 1) [204]. Additionally, cells can regulate the ROS generated by Fenton reaction indirectly through the FTH, which mediates the suppression via LIP sequestration (Figure 6) [205], which in turn reduces ROS production. Surprisingly, cancer cells may be able to adapt to the toxicity caused by ROS after receiving a high-dose of ascorbate, by upregulating the intracellular antioxidant proteins and non-enzymatic molecules.

SODs are potent metalloenzymes found in all living organisms that catalyze the transition of O_2_^•−^ into O_2_ and H_2_O_2_ by alternating oxidation-reduction of metal ions existing in the active site of SODs; thus, preventing the harmful effects of ROS. Based on the metal co-factors located in the active sites, SODs are classified as Manganese-SOD (Mn-SOD), Iron-SOD (Fe-SOD), Nickel-SOD, and Copper-Zinc-SOD (Cu, Zn-SOD) [206,207]. In cancer, for instance, Mn-SOD was found to play the role of a double edged sword agent, as this protein was differentially expressed in different tumors [207,208]. In breast cancer, Mn-SOD gene expression is drastically altered between early and advanced stage, in such a way that it is decreased in the early breast cancer stage and increased in the advanced stage. Furthermore, via suppressing O_2_^•−^ and H_2_O_2_, this protein was linked to the invasive and angiogenic characteristics of breast tumor cells [209]. However, Mn-SOD in cancer development is still controversial, as it can be considered as a protective antioxidant, as well as a pro-oxidant during cancer progression. 

CAT is an important enzyme of the peroxisomes that metabolizes H_2_O_2_ to form H_2_O and O_2_, as a form of defense against oxidative stress [210]. CAT is absent in the mitochondria; therefore, GPx mediates the reduction of H_2_O_2_ to H_2_O, and of lipid peroxides to their corresponding alcohols [202]. In cancer, CAT serves to protect malignant cells from intercellular ROS damage, given that the ability to withstand ROS action is essential for tumor development [211]. Expression of CAT has also been shown to vary between cancerous and non-cancerous tissues. The protein is expressed differentially in various malignancies as well [212].

Glutathione peroxidases (GPxs) are a group of proteins that use GSH as a co-substrate to reduce H_2_O_2_ to H_2_O, and lipid peroxides to their corresponding alcohols [202,213,214]. Notably, because they need selenium as a co-factor for their action, GPxs are classified as seleno-cysteine peroxidase [213]. In cancer, these proteins can serve as both protective antioxidants, as well as pro-oxidants. For example, in CRC, GPxs was found to reduce cancer growth by acting as a tumor suppressing gene [215], while in breast cancer, high expression of GPx was found to develop resistance to chemotherapy, leading to a poor response to treatment in breast cancer patients [216].

Peroxiredoxins (PRDXs) are a widespread group of thiol-dependent peroxidases that catalyze the conversion of H_2_O_2_, alkyl hydroperoxides, and peroxynitrite, and alkyl hydroperoxides, and peroxynitrite to H_2_O, and the corresponding alcohol and nitrite [217]. Moreover, PRDXs have been shown to have a low or high expression in cancer in several investigations. Several in vitro and in vivo investigations have found that high levels of PRDX expression may either prevent or stimulate tumor development [217], depending on the type of cancer, the stage of the disease, and the PRDX family member [217,218,219].

Thioredoxins (TRXs) are a broad family of small dithiol proteins that share the thioredoxin fold motif [220,221] responsible for reducing the oxidized cysteine residues on cellular proteins [222]. As part of its disulfide reductase activity and manipulation of the protein dithiol/disulfide balance, NADPH, thioredoxin, and TrxR are all components of the thioredoxin system, which is a key defense mechanism against ROS [223]. In cancer, Thioredoxin-1 (Trx-1), for example, is expressed in great quantities in several malignancies and has been linked to worse patient survival rates, in addition to resistance to chemotherapy [224]. Furthermore, the protein enhances VEGF expression in vitro and angiogenesis in vivo, through regulating and increasing transcription factors such as HIF1-α [225].

Reduced glutathione (GSH) is the most prevalent low-molecular-weight non-enzymatic thiol linear tri-peptide comprising L-glutamine, L-cysteine, and glycine molecules in living organisms, and it plays an important role in redox homeostasis, which protects against the oxidative stress caused by ROS [226]. Importantly, through enzymatic reactions, GSH directly and indirectly scavenges free radicals and other ROS, as well as reactive nitrogen species (RNS) [227]. Since it includes N-L-gamma-glutamyl-cysteinyl glycine or L-glutathione, the protein has a sulfhydryl (SH) group on the cysteinyl side, which contributes to the strong electron-donating properties of the protein. Under normal physiological conditions, GSH is present in its reduced form, and its oxidation to create oxidized GSSG is mediated by interactions with free radicals and/or antioxidant enzymes, such as GSH peroxidases, which use GSH as a cofactor to reduce H_2_O_2_ and phospholipid hydroperoxide [228]. However, glutathione reductase reduces GSSG via a NADPH-dependent mechanism, to keep the intracellular concentration of GSSG at extremely low concentrations [229].

In cancer cells, GSH may act as a protective factor against the ROS produced by high-dose Vit-C. For instance, it has been suggested that increased GSH levels and the rate of GSH production in malignant cells are responsible for the resistance to chemotherapy and radiation therapy [228,230]. In addition, research conducted on DU-145, a human prostate cancer cell line, showed that higher GSH levels were associated with drug resistance. Similar results were also reported in ovarian cancer [228].

Glutathione-S-transferases (GSTs) are a superfamily of abundant enzymes (α, μ, ω, π, θ, and ζ) that protect cellular components from the oxidative stress caused by ROS [231]. GSTs promote the conjugation of GSH to various endogenous and exogenous electrophiles, and this represents the first step in the elimination of toxic compounds by the mercapturic acid pathway [232], resulting in the formation of less reactive and more soluble molecules. There is a growing body of evidence that suggests that GST overexpression plays a vital role in cancer resistance and progression. Drug resistance may be triggered by GSTs in two ways: either directly via detoxification, or by inhibiting the MAP kinase pathway [232]. Furthermore, GST knockdown inhibited cancer development, suggesting that an increase in ROS levels may be related to impaired cell proliferation and growth [233].

FTH, ferritin is the main iron storing protein in the cell and is composed of 24 peptide chains divided into two subunits: FTH, and ferritin light chain (FTL) [81]. These proteins come together to create an iron-sequestering complex. Through its ferroxidase activity, FTH converts excess cellular iron into the harmless ferric ion, which is then stored in a ferritin complex; thereby, preserving proper iron homeostasis [81]. Moreover, FTH functions as a regulator that sequesters the LIP into low level concentrations. FTH was shown to be an important facilitator of the antioxidant and protective properties of NF-κB. FTH is induced downstream of NF-κB and is necessary to avoid persistent JNK activation and, hence, the apoptosis produced by TNF-α. The suppression of JNK signaling by FTH is dependent on the prevention of ROS production via sequestration of LIP [205]. FTH overexpression in cancer cells may lead to resistance to high-dose Vit-C therapy, which may induce massive ROS release. Kiessling et al. reported that in this scenario, since FTH is activated downstream of NF-κB, downregulation of this transcription factor in cutaneous T-cell lymphoma results in reduced expression of FTH, which leads to an increase in LIP, causing huge formation of ROS [174]. Conversely, siRNA-mediated direct downregulation of FTH resulted in ROS-dependent apoptosis. However, T cells from healthy subjects do not demonstrate FTH downregulation and, hence, do not show an increase in iron or cell death when NF-κB is inhibited. Furthermore, in breast cancer cell lines, FTH overexpression was related to modifications in the subcellular location of these molecules, as shown by an increased level of nuclear ferritin and a reduced level of the LIP [234], which affected ROS production. 

Overexpression of ROS-scavenging systems in TME might be associated with cancer development and progression, and these systems could have a direct impact on the toxicity caused by high-dose Vit-C therapy, which is heavily reliant on free radicals. Furthermore, overexpression of FTH, which facilitates the sequestration of the LIP, may indirectly impact the generation of ROS caused by high-dose Vit-C. Moreover, cancer cells may overexpress many scavenging systems, which are occasionally expressed in a subservient manner. To improve the efficacy of this therapy, studies investigating the cancer-specific expression of these systems, in terms of applying high-dose Vit-C, are strongly encouraged. Studies to elucidate the molecular pathways that can be employed by these systems, to give a greater protection against the oxidative stress and that can be exploited by specific tumors, are also strongly suggested.

**Table 1:** The function of defense systems and their sub-cellular localizations.

Defense System	Sub-Cellular Localization(s)	ROS Type and System Function	Ref.
SODs	Cytosol and Peroxisomes	O_2_^•−^ to O_2_ and H_2_O_2_	[202]
CAT	Peroxisomes	H_2_O_2_ to H_2_O and O_2_	[202]
GPxs	Mitochondria and Cytosol	H_2_O_2_ to H_2_O lipid peroxides to alcohols	[202]
PRDXs	Cytosol, Mitochondria, Nucleus And Endoplasmic reticulum	H_2_O_2_ to H_2_O	[217]
TRXs	Cytosol, Mitochondria, Nucleus	H_2_O_2_ to H_2_O and O_2_	[220,221]
GSH	Cytosol, Mitochondria, Nucleus And Endoplasmic reticulum	H_2_O_2_ to H_2_O	[227,229]
GSTs *	Cytosol, Membrane-bound	conjugates with reduced GSH)	[232]
FTH *	Nucleus, Lysosome, Cytoplasm	Sequester Fe(II) to inhibit ROS generation	[81]

SODs, superoxide dismutases; CAT, catalase; PRDXs, Peroxiredoxins; GSTs, glutathione-S-transferases; TRXs, Thioredoxins; GPxs, glutathione peroxidases; GSH, reduced glutathione; GSSG; FTH, ferritin heavy chain, indirect ROS formation inhibition *.

## 9. Clinical Studies of High-Dose Vitamin-C in Cancer Therapies

Over the last decade, there has been a rise in the number of phase I/II clinical trials and case reports evaluating the safety and effectiveness of high-dose ascorbate, as a monotherapy or in combination with radiation and other traditional chemotherapies for different cancer types, such as ovarian, brain, prostate, and lung cancers [235,236,237]. Wang et al. found that, in a phase I clinical study of patients with metastatic gastric (mGC) and metastatic colorectal (mCRC) cancers, a high dose of i.v ascorbic acid (1000 mg/day) in combination with other anti-cancer agents (mFOLFOX6 or FOLFIRI) demonstrated a potentially improved efficacy, with appropriately reduced side effects; thus, improving the patients’ quality of life [238]. Another study reported the use of oral Vit-C (1000 mg/d) supplementation in conjunction with neo-adjuvant chemo-radiation (40 Gy, cisplatin) for esophageal adenocarcinoma (EC). After oral Vit-C pretreatment, NF-κB activity and cytokines were activated in cancer tissue. In 25% of subjects, NF-κB activity was downregulated after the therapy, including two cases from the Vit-C arm. The cancer group had a considerable drop in cytokine levels, which was much more obvious in the Vit-C cohort [239]. An additional study examining the combined effect of i.v Vit-C (1000 mg/d) and chemotherapy (Gemcitabine, carboplatin) on triple-negative breast cancer (TNBC) discovered that some cases experienced complete remission (CR), while other cases experienced partial remission (PR) and stable disease (SD) after treatment, when compared to the control group. The frequency of adverse events in the treatment group was substantially lower than in the control group, indicating that Vit-C may have an impact on improving the prognosis of patients with advanced TNBC [240]. 

Similarly, patients with myeloma malignancy were given arsenictrioxide/bortezomib/Vit-C (1000 mg/d)/dexamethasone (ABCD) and bortezomib/dexamethasone (BD) regimens. The individuals treated with ABCD had a better response than patients treated with BD. In addition, as compared to the BD regimen, ABCD was a more effective and tolerable regimen for newly diagnosed myeloma patients [241]. Another report evaluated the effect of alkalization treatment and i.v Vit-C (25,000–50,000 m/d) on chemotherapeutic outcomes in patients with small-cell lung cancer (SCLC). The patients were divided into two groups: those who received alkalization therapy and intravenous Vit-C treatment in addition to standard chemotherapy, and those who received chemotherapy alone. The study showed that the intervention group’s median overall survival was 44.2 months, whereas the control group’s was 17. These data suggest that combining alkalization therapy and intravenous Vit-C therapy may improve outcomes in SCLC patients receiving chemotherapy [242]. 

According to the results of a randomized controlled study performed on patients with myeloid malignancies, oral Vit-C (500 mg/d) supplementation combined with DNMTi-treatment (5-azacytidine, 100 mg/m^2^/d for 5 days in 28-day cycles) restored the plasma Vit-C concentration levels to normal. As a consequence, the global levels of 5hmC/5mC in mononuclear myeloid cells from patients increased significantly. Despite these promising findings, the clinical efficacy of this combination should be investigated in a large randomized, placebo-controlled clinical trial [191].

On the contrary, research on colon cancer patients found that administering high-dose Vit-C (1000–25,000 mg/d) through i.v infusions for four days before surgical resection resulted in significantly higher plasma concentrations and compartmentalization of these high Vit-C concentrations into erythrocytes. Furthermore, ascorbate levels rose across the board in all tumor areas, indicating that enhanced Vit-C plasma availability enabled efficient accumulation throughout the tumor. In addition, tumor hypoxia indicators were reduced in post-infusion tumors compared to the control group, suggesting that raising Vit-C levels may attenuate the activation of hypoxia-inducible factors. However, there were no major side effects, and the ascorbate infusion had no effect on the patients’ quality of life [30].

## 10. Conclusions and Future Prospects

Regardless of the fact that ascorbic acid has been shown to have anticancer properties for more than 50 years, most of the mechanisms behind this action have remained a mystery. However, new results have advanced our knowledge of the biological roles and processes behind Vit-C’s anti-cancer activity; emphasizing a number of fascinating and interesting notions that offer solid scientific foundations for the use of Vit-C in cancer treatment. Ascorbate is essential for the activity of cancer-related proteins such as NF-κB, HIFs, GLUT-1, TETs, FIHs, and PHDs, all of which are involved in cancer progression and development. The discovery of ascorbate-regulated pathways will allow the development of therapeutic strategies to sensitize tumor cells to the use of ascorbic acid. Moreover, since ascorbate mediates its action through the generation of massive amounts of ROS, ascorbate oxidation requires the recycling of Fe(II)/Fe(III), where FTH sequesters the Fe(II) required in the production of ROS via the Fenton reaction. Not many studies have focused on the utilization of Cu(II)/Cu(I) to induce ascorbate oxidation, and it would be interesting to investigate if the absence of Fe(II) could be compensated for by Cu(II)/Cu(I) oxidizing ascorbate instead. Indeed, tremendous effort has been made in understanding the processes behind the redox control of the NF-kB pathway during the recent decades. More research into this crucial area will aid our understanding of how the NF-κB pathway and oxidative stress interact with other cellular signaling networks. The development of therapeutic options for the treatment of cancer might be aided by a better knowledge of the complicated interaction between the NF-κB pathway and oxidative stress, particularly that mediated by high-dose Vit-C.

Furthermore, high-dose Vit-C has shown great promise in improving cancer immunotherapy efficacy, particularly when combined with ICP, via immune system activation and cancer cell sensitization to therapy, in a variety of in vivo and in vitro studies. However, to date, no clinical trials have employed this essential strategy, necessitating additional investigations. In fact, it is critical to investigate whether high-dose Vit-C, in addition to its function in activating immune cells, may be used to minimize the toxicity caused by the combination of at least two ICP therapies. In addition, future research should concentrate on defining the precise combination dose that is likely to be efficient, less hazardous, and practicable for use in the treatment of cancer patients suffering from a variety of malignancies. 

Since high-dose Vit-C can induce massive ROS concentrations that can damage cancer cells, these cells, in return, can possess highly effective antioxidant systems that can easily scavenge ROS and reduce their toxic effect. In fact, a single cancer cell can contain all of these systems, which may act in a complementarity manner to reduce the deleterious effect of ROS. Thus, a significant effort should be made to comprehend the mechanisms behind redox regulation by antioxidant systems. More studies into this critical area will help us understand how these antioxidant systems interact with one another and with other cellular signaling networks. A deeper understanding of the intricate interplay between the numerous antioxidant systems and how they work together to minimize the impact of high-dose Vit-C may assist in the development of therapeutic approaches for cancer treatment.

Moreover, these contentious data suggest that there is no benefit to intravenous delivery of Vit-C over oral treatment. However, more clinical trials evaluating the optimal method of high-dose Vit-C delivery are urgently required.

Finally, high-dose Vit-C has the definite potential to provide beneficial and cost-effective anti-cancer treatment options that should be investigated further. Ascorbic acid may become a significant treatment option in the fight against cancer, due to its widespread availability in nature, minimal toxicity, and low cost.

## Data Availability

Not applicable.
